# Bilateral Primary Breast Cancer With Discordance in Molecular Subtypes: A Case Report

**DOI:** 10.1155/ijbc/9082803

**Published:** 2025-06-23

**Authors:** Yingying Rao, Qian Zhan, Hengyu Li

**Affiliations:** ^1^Department of Breast and Thyroid Surgery, The First Affiliated Hospital of Naval Medical University, Shanghai, China; ^2^Department of Imaging, The First Affiliated Hospital of Naval Medical University, Shanghai, China

## Abstract

Molecular subtype is a crucial prognostic factor for bilateral breast cancer and plays a key role in guiding treatment decisions. Several studies have confirmed that the expression patterns of hormone receptor and human epidermal growth factor receptor 2 are generally consistent in synchronous bilateral primary breast cancer. Discordance in the receptor expression status is commonly associated with poorer prognosis in synchronous bilateral breast cancer. However, there is currently limited literature reporting such cases. This article presents a case of synchronous bilateral primary breast cancer with discordant molecular subtypes and reviews relevant literature.

## 1. Introduction

Bilateral primary breast cancer (BPBC) is a rare form of breast cancer characterized by the occurrence of independent primary cancer lesions in both breasts. Incidence rates of BPBC reported in the literature range from approximately 1.4% to 11.8% [1]. Based on the time interval between occurrences, BPBC is further categorized into synchronous bilateral breast cancer (SBBC) and metachronous bilateral breast cancer (MBBC) [1–3]. Most studies classify BPBC occurring within 6 months as SBBC, while those beyond 6 months are categorized as MBBC [2]. However, there is currently no consensus on the time interval cutoff for differentiating between these two breast cancer subtypes [1, 4].

Molecular subtypes, as crucial prognostic factors for early-stage breast cancer, are commonly used to guide treatment decisions [5, 6]. For BPBC, most current research supports a tendency toward consistent intertissue receptor expression, with SBBC exhibiting a higher consistency rate in molecular subtypes compared to MBBC [2, 7–9]. This may be attributed to similar hormonal influences and tumor microenvironments during SBBC development, whereas systemic treatment for the initial cancer in metachronous cases may alter the biomarker status of the second primary cancer, thus leading to a lower consistency rate in MBBC molecular subtypes [2]. An estimated 5% of SBBC cases exhibit discordance in receptor status between the two primary breast cancers, which is associated with poorer prognosis and treatment challenges [1, 10, 11].

Given the rarity of SBBC cases with discordance in molecular subtypes, this article is aimed at providing a detailed case analysis in order to facilitate a deeper understanding of these complex cases.

## 2. Case Report

**Figure 1:**
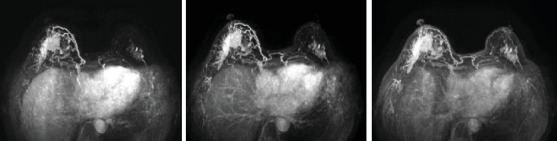
MRI contrast-enhanced dynamic imaging (early, delayed, and late phases) demonstrates diffuse heterogeneous enhancement in the outer quadrant of the right breast, thickening of the right internal mammary artery, and focal heterogeneous enhancement in the outer quadrant of the left breast. All enhancements demonstrate arterial inflow characteristics. BI-RADS V.

In September 2013, a 59-year-old postmenopausal woman was admitted to our hospital. Her primary complaint was bilateral breast masses, initially detected 2 months prior to presentation. Upon admission, physical examination revealed a 3-cm surgical scar in the upper outer quadrant of the right breast, accompanied by a painless, firm mass measuring approximately 4 × 4 cm in the same quadrant. In the left outer upper breast quadrant, a painless, flaky mass about 3 × 2 cm in size was found. The patient's medical history review indicated a prior total hysterectomy and right breast lesion excision. These procedures, performed several years earlier, were indicated for uterine fibroids and right mammary adenosis. The patient reported no family history of breast or ovarian cancer. After admission, the patient underwent breast ultrasound, x-ray, and MRI examinations. The results indicated BI-RADS scores of V (see Figure 1). Nodular lymph nodes were seen in both armpits, with the largest measuring about 0.9 cm in diameter on the right side. Chest radiographs and other relevant examinations showed no significant abnormalities.

Combined with the results of the bilateral breast clinical physical examination, imaging data, preoperative biopsy of the mass, and intraoperative frozen section pathology, the patient underwent bilateral modified radical mastectomy. Postoperative pathological results showed Grade II infiltrating ductal carcinoma in the right breast with axillary lymph node metastasis (15/15) and invasive lobular carcinoma in the left breast with no axillary lymph node metastasis (0/19). Immunohistochemistry (IHC) of the right tumor showed ER (−), PR (60%), CerbB2 (++), and Ki-67 (50%) in the right tumor, while the left tumor showed ER (95%), PR (95%), CerbB2 (+), and Ki-67 (5%) in the left tumor. Additionally, HER-2 gene amplification was confirmed in the right tumor by FISH. Postoperative PET-CT showed no significant abnormalities.

The selection of postoperative adjuvant treatment follows the strategy of “high-risk side guidance, combined coverage, and dynamic adjustment.” Per *NCCN guidelines* [12], the patient's right-sided tumor is high-risk (high proliferation index, multiple lymph node metastases) and HER2-positive, requiring intensive chemotherapy combined with trastuzumab-targeted therapy. Adjuvant endocrine therapy is recommended for the left-sided HR-positive tumor. Given the bilateral molecular subtype discordance, we recommend postoperative intensive chemotherapy with targeted therapy (epirubicin/cyclophosphamide followed by docetaxel/trastuzumab). This regimen primarily targets the high-risk right-sided tumor while leveraging chemotherapy's broad-spectrum effects to suppress potential bilateral micrometastases. The left-sided hormone receptor–positive tumor requires long-term endocrine therapy (anastrozole), which may also address potential hormonal sensitivity in the right-sided tumor. The treatment plan will be dynamically adjusted based on ongoing efficacy evaluations. The patient showed good tolerance to the postoperative adjuvant treatment, with no significant abnormalities found in routine blood tests, liver and kidney function indices, or echocardiograms.

**Figure 2:**
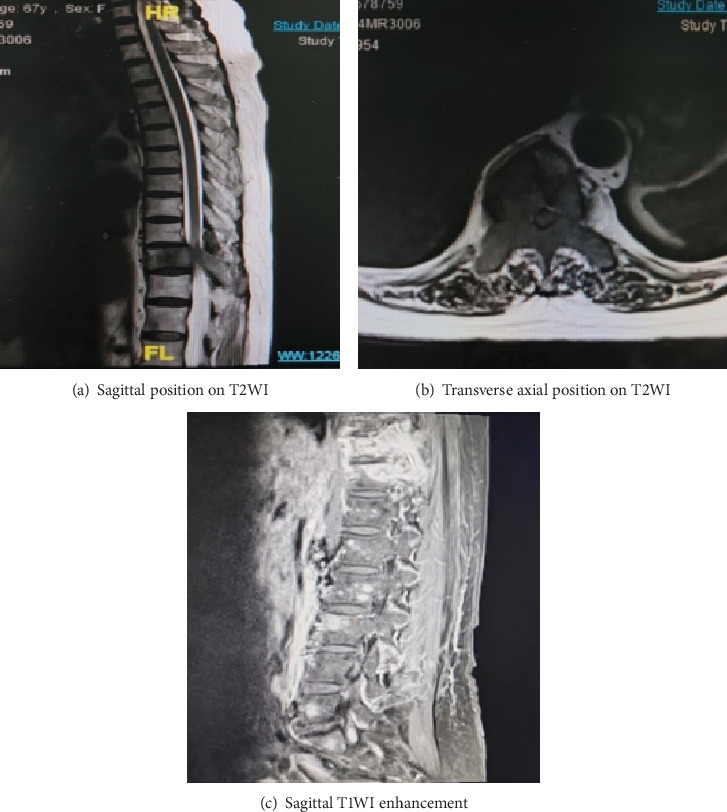
MRI imaging from June 2022. (a) Sagittal position on T2WI indicated decreased T10 vertebral signal, mild deformation, poor T9/10 alignment, local spinal stenosis, and acceptable upper and lower intervertebral discs. (b) Transverse position on T2WI indicated diffuse reduction of signals in the T10 vertebrae and annexes and slight swelling of local bone structure. (c) Sagittal T1WI enhancement indicated multiple lamellar enhancement foci in thoracolumbar vertebrae and adnexa, with a focus on the T10–11 vertebrae.

**Figure 3:**
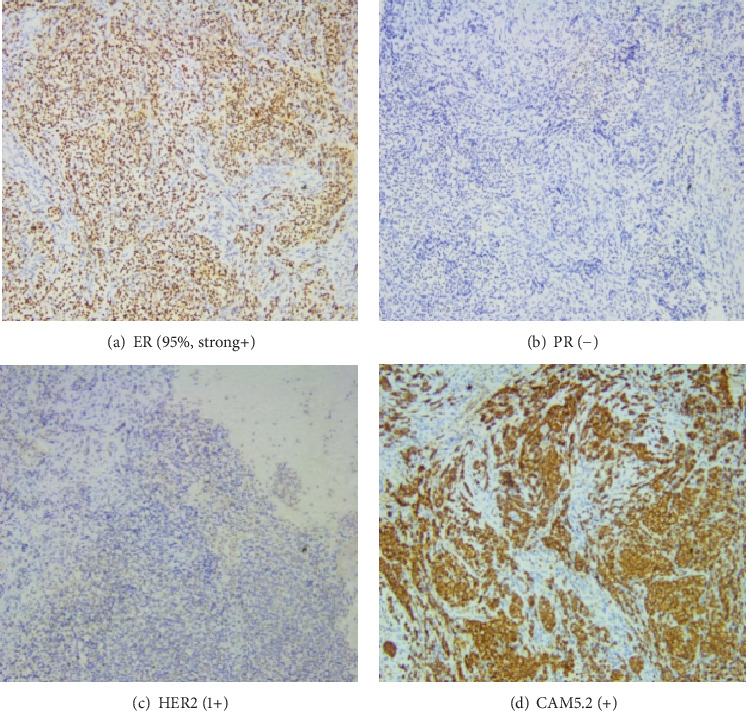
Immunohistochemistry results from the posterior thoracic puncture biopsy of the manifestations at the T10 level showing ER (95%, strong+), PR (−), HER2 (1+), and CAM5.2 (+), consistent with metastatic breast cancer.

**Figure 4:**
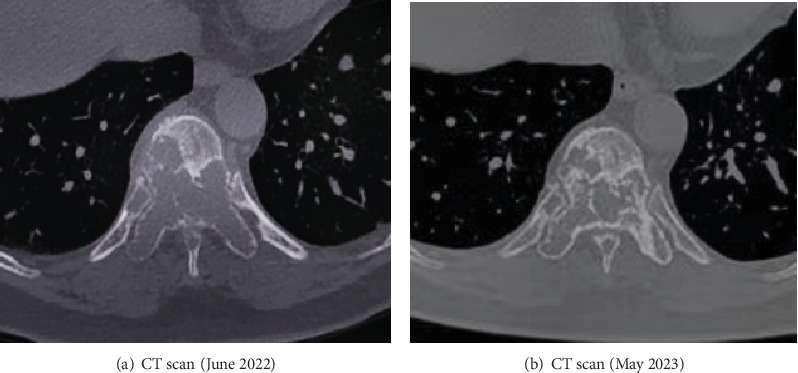
Comparison of CT bone window at the same level from June 2022 and May 2023. In June 2022, (a) CT bone window showed local bone trabecular destruction of the T10 vertebral body, accessories and adjacent ribs, and slight swelling changes in local bone structure. (b) CT bone window from May 2023 revealed bone density deposits in the damaged area of the original bone trabecula.

**Figure 5:**
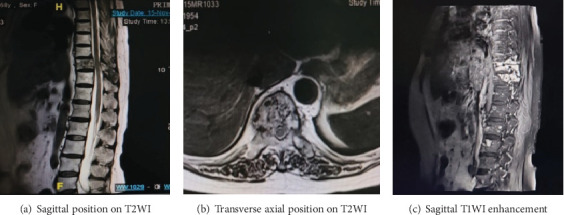
MRI imaging from November 2023. (a) Sagittal position on T2WI indicated uneven signals in the T10 vertebral body and local spinal stenosis, which had improved. (b) Transverse position on T2WI indicated uneven signals in the T10 vertebral body and accessories and clear signals in local bone. (c) Sagittal T1WI enhancement indicated multiple enhancement foci in the thoracolumbar vertebra and the adnexa, narrowing the scope of the anterior lesion.

In June 2022, the patient was readmitted with complaints of recurrent lower back pain, accompanied by right lower limb pain and weakness persisting for 6 months and an inability to lie flat for the past 4 months. Bone scan and thoracolumbar MR revealed multiple osteolysis metastases in the T9-S2 vertebra with some appendages, significant bone damage in T10–11, spinal canal stenosis at the T10 level, and thoracic cord compression (see Figure 2). Posterior thoracic puncture biopsy revealed ER (95%, strong+), PR (−), HER2 (1+), and Ki67 (5%) manifestations at the T10 level (see Figure 3). Considering the primary tumor condition and IHC results of bone metastases, the patient started first-line endocrine therapy with fulvestrant in combination with abemaciclib in August 2022, along with bone protection treatment with denosumab. After 1 month of treatment, the patient reported significantly reduced limb pain, and by 2 months, the patient could lie flat, indicating improved quality of life. Follow-up CT and MRI in 2023 showed limited bone destruction and evidence of bone repair (see Figures 4 and 5).

As of March 2024, the patient was still receiving combination therapy with abemaciclib, fulvestrant, and denosumab. Her condition remained stable, with no significant findings in recent follow-ups.

Tables 1 and 2 summarize the patient's clinical history, treatment regimens and timelines, and therapeutic efficacy evaluations.

## 3. Discussion

Studies have shown that SBBC has distinct characteristics compared to unilateral breast cancer and MBBC. Patients with SBBC tend to be older and have a higher ER-positive rate and a higher incidence of lobular cancer and are more likely to undergo mastectomy than those with unilateral breast cancer. Younger age at onset, family history of breast cancer, and bilateral breast cancer with discordance in molecular subtypes are associated with poorer clinical outcomes. In addition, patients with bilateral ER-negative disease are more likely to have BRCA1/2 mutations [1, 13, 14]. At present, there are no standard treatment guidelines for SBBC. The presence of simultaneous primary tumors with different receptor expression states may further complicate the disease, affecting the treatment response, risk of recurrence, and prognosis [15–18].

The discordance in molecular subtypes of SBBC is characterized by differences in hormone receptor and HER2 receptor expression status. Special attention should be paid to ER expression in hormone receptor status, as it holds high clinical value in assessing prognosis. Studies have shown that SBBC patients with inconsistent ER expression between the two primary breast cancers had a higher mortality rate than those with consistent ER expression during the first 5 years of monitoring [19, 20]. In the present case, the patient had consistent PR expression status, discordant ER and HER2 status, and different histological types between the two primary lesions. Following bilateral modified radical mastectomy and postoperative adjuvant therapy, the patient achieved approximately 104 months of recurrence-free survival. At present, the first-line treatment for bone metastasis has effectively controlled disease progression and improved the quality of life. Subsequent treatment and follow-up are ongoing.

A review of the treatment process of this patient reveals several points worthy of consideration.

1. According to the Chinese expert consensus statement on the clinical diagnosis and treatment of breast cancer bone metastasis and bone-related disease [21], it is recommended that postmenopausal patients with early-stage breast cancer receiving aromatase inhibitors use bone-modifying agents (bisphosphonates or denosumab) to effectively prevent bone loss caused by anticancer treatment. The use of bisphosphonates in the adjuvant treatment of early-stage breast cancer can reduce the risk of recurrence and provide survival benefits [22]. For this patient, early postsurgery use of bone-modifying agents could potentially extend disease-free survival and bring more clinical benefits.
2. The patient's tumor on the right side shows lymph node metastasis (+), ER (−), and PR (60%), while on the left side, it is HR (+) with no lymph node metastasis. This observation raises the controversial issue of whether ER (−) and PR (+) breast cancer typing is an artifact of IHC hormone receptor detection or represents a distinct breast cancer subtype. Literature reports suggest that among patients with single positive hormone receptors, those with ER (−) and PR (+) disease accounted for a small proportion (about 1%–4%), and PAM50 molecular typing classifies the majority of these cases as triple-negative (53%–65%), followed by luminal type (15%–27%) [23]. This suggests that most of these breast cancer cases share the clinical features and prognosis of triple-negative breast cancer, while luminal breast cancer is more sensitive to endocrine therapy. In the present case, the patient received abemaciclib in combination with endocrine therapy, a regimen investigated in the MonarchE study. In the MonarchE study, adjuvant abemaciclib demonstrated sustained benefit in reducing the risk of recurrence and distant metastasis in hormone receptor–positive, HER2-negative, node-positive, high-risk early breast cancer [24]. Given that patients with ER-positive status accounted for the vast majority of the enrolled population, the potential use of PR status in guiding treatment decisions and prognostic assessments for ER (−) and PR (+) cases warrants consideration. This also raises the question of whether patients with ER (−) and PR (+) tumors may also benefit from postoperative CDK4/6 inhibitors combined with endocrine therapy. Assuming the exclusion of technical detection errors, if the CDK4/6 inhibitor (abemaciclib) had been approved, patients might experience a more significant progression-free benefit from combining abemaciclib with endocrine treatment for postoperative adjuvant therapy?
3. Patients with breast cancer bone metastasis experiencing bone-related complications such as bone pain often benefit from treatments like radiotherapy and orthopedic surgery, aimed at alleviating symptoms, improving quality of life, and prolonging survival. In this case, the patient presented with bone pain and spinal cord compression, qualifying her for radiotherapy and orthopedic surgery due to potential spinal instability indicated by Tomita, Tokuhashi, and spinal stability scores. However, considering the favorable drug response and prognosis indicated by ER (95%, strong+) and HER2 (−) biopsy results, particularly in the stable thoracic vertebrae with severe metastatic lesions, endocrine therapy was proposed as an alternative to avoid unnecessary surgery and reradiotherapy. The patient opted for endocrine therapy with abemaciclib combined with fulvestrant and achieved significant symptom reduction as well as quality-of-life improvement within 2 months after treatment initiation. Subsequent follow-up examinations revealed alleviation of spinal cord compression and containment of bone destruction, highlighting the importance of considering pathological subtypes, disease progression, and prognosis in comprehensive treatment strategies for patients with breast cancer bone metastasis. Repeat biopsy and dynamic monitoring of receptor expression status in metastatic lesions are essential for tailoring individualized treatment plans. In addition, multidisciplinary team collaboration can facilitate more comprehensive efficacy assessment, especially in bilateral breast cancer cases with discordance in molecular subtypes or metastasis, ultimately improving clinical outcomes.
4. Bone metastases are typically considered unmeasurable due to the difficulty in directly quantifying their response to treatment, presenting significant clinical challenges in assessing efficacy. The assessment of bone metastasis treatment response is based on a combination of imaging tests, biochemical markers, symptom assessment, and bone-related events. In this case, the patient's most severe site of bone metastasis was at the T10 level. Following treatment with fulvestrant in combination with abemaciclib and denosumab, there were notable changes in bone density and CT values observed through CT scans (bone window). The CT value increased from 47.4 HU at diagnosis in June 2022 to 812.6 HU by May 2023, representing the average from the area showing the most significant bone repair. CT scans offer a more direct and accurate method for assessing the effectiveness of treatments for breast cancer bone metastases. Changes in CT values may provide a quantitative basis for bone repair and guidance for subsequent treatments [25]. Denosumab is commonly used as a bone-protective treatment, effectively preventing bone-related adverse events, alleviating pain, and improving quality of life. In this case, following denosumab treatment, the patient experienced significant pain relief, improved quality of life, and osteoblastic bone healing. Denosumab is administered via subcutaneous injection once every 6 months, offering a convenient dosing schedule that enhances treatment compliance. To date, the patient has maintained regular denosumab treatment and has not experienced any other bone-related adverse events.
5. As previously noted, BPBC with discordant molecular subtypes can significantly impact treatment decisions and holds critical clinical importance, necessitating further exploration of the underlying molecular mechanisms. Existing literature suggests that potential mechanisms include clonal heterogeneity, epigenetic regulation, and genetic drivers [26, 27]. First, bilateral breast cancers may arise from distinct progenitor cell clones within the breast, resulting in divergent molecular subtypes and histological features [26]. Furthermore, epigenetic mechanisms such as DNA methylation, histone modification, and noncoding RNA may selectively regulate hormone receptor or HER2-related genes, indicating that epigenetic dysregulation contributes to the development of distinct molecular subtypes [27]. Finally, genetic predisposition and somatic mutations may disrupt genomic stability, thereby contributing to tumor heterogeneity in bilateral cases [26]. In summary, the molecular mechanisms underlying discordant subtypes in BPBCs are multifactorial. Future research should integrate multiomics approaches (e.g., single-cell sequencing) to unravel these complexities and guide precision therapy.

To sum up, standard treatment guidelines for SBBC are still lacking, and there is limited literature reporting SBBC cases with discordant molecular subtypes. Bilateral mastectomy remains the primary surgical treatment, and postoperative adjuvant treatment should refer to the primary lesion at a more advanced stage [28–31]. When managing this patient group, clinicians should comprehensively consider tumor histological type, disease stage, bilateral lesions, and molecular typing of metastatic lesions to tailor individualized treatment plans. Close monitoring during follow-up is essential to evaluate efficacy and make comprehensive judgments.

## 4. Conclusions

The individualized treatment of BPBC, especially with discordant molecular subtypes, lacks standardized guidelines. This SBBC case with molecular discordance underscores the need for personalized treatment and a multidisciplinary approach. Following bilateral modified radical mastectomy, the patient received adjuvant EC-TH and endocrine therapy. Upon recurrence with bone metastases, a regimen of abemaciclib, fulvestrant, and denosumab was implemented, effectively controlling disease progression. For patients with discordant molecular subtypes in bilateral breast cancer, bone biopsy is recommended upon bone metastasis to accurately assess pathological typing, guiding personalized treatment plans. If bone metastasis is confirmed, bone-modifying agents should be consistently prescribed to reduce the risk of skeletal-related events (SREs) and improve quality of life. Subsequent efficacy evaluations should integrate symptom assessment, imaging, biochemical markers, and bone-related events. Changes in CT values may quantitatively indicate bone repair and guide further treatment. This article presents a case of SBBC with molecular discordance, reviewing its diagnosis and treatment. Future exploration by breast specialists for such patients should focus on providing precise treatment strategies and maximizing clinical benefits for patients.

## Figures and Tables

**Table 1:** Patient history review.

**Category**	**Details**	
Brief history		
Gender	Woman	
Age at first visit (years)	59	
Chief complaint	Bilateral breast masses detected 2 months prior to admission	
Past medical history	Uterine fibroids, right mammary adenosis	
	Total hysterectomy	
	Right breast local lesion excision	
Obstetric history	Married with one child	
Family history	Denied family history of breast and ovarian cancer	
Operation (2013-09)	Modified radical surgery for bilateral breast cancer	
Pathological diagnosis	Right breast: Invasive ductal carcinoma, pT2N3M0	
	Left breast: Invasive lobular carcinoma, pT2N0M0	
	Vertebral bone metastasis (T10-S2) (2022-06)	
Molecular subtyping	Right breast: ER(−), PR (60%), HER2 (++)	
	HER2 FISH amplification (+), Ki-67 (50%)	
	Left breast: ER (95%), PR (95%), HER2 (+), Ki-67 (5%)	
	Bone metastases: ER (95%, strong+), PR (−), HER2 (1+), Ki-67 (5%)	

Postoperative		
2013-10~2014-12	Adjuvant therapy	EC-TH regimen
	Radiotherapy	Right chest wall radiotherapy (50 Gy/25 F)
		Right supraclavicular radiotherapy (50 Gy/25 F)
2014-03~2022-06	Endocrine therapy	Anastrozole
2022-08~2024-03	First-line treatment	Abemaciclib + fulvestrant + denosumab

**Table 2:** Therapeutic effect evaluation.

**Timeline**	**Evaluation methods**	**Results**	**Remarks**
2013-09~2021-09	Routine tests	NED	No residual lesions postsurgery
	Ultrasound, CT/PET-CT		No distant metastases on imaging
2022-06	ECT, MRI, CT + biopsy	PD^a^	New T10-S2 vertebral metastases confirmed by biopsy
			Bone pain symptoms
2022-08~2023-05	Clinical symptoms	SD	Nonmeasurable lesion evaluation
	CT, MRI		Symptomatic improvement
			Osteoblastic changes on imaging
			No new lesions
2023-05~2024-03	Routine tests	SD	Partial osteoblastic remodeling
	CT, MRI		Stable imaging findings without disease progression

Abbreviations: NED, no evidence of disease; SD, stable disease per RECIST 1.1.

^a^PD, per clinical progression with new symptomatic bone lesions.

## Data Availability

The data that support the findings of this study are available from the corresponding author upon reasonable request.

## References

[1] Jiang H., Zhang R., Liu X. (2021). Bilateral Breast Cancer in China: A 10-Year Single-Center Retrospective Study (2006–2016). *Cancer Medicine*.

[2] Ding S., Sun X., Lu S., Wang Z., Chen X., Shen K. (2021). Association of Molecular Subtype Concordance and Survival Outcome in Synchronous and Metachronous Bilateral Breast Cancer. *Breast*.

[3] Sakai T., Ozkurt E., DeSantis S. (2019). National Trends of Synchronous Bilateral Breast Cancer Incidence in the United States. *Breast Cancer Research and Treatment*.

[4] Holm M., Tjønneland A., Balslev E., Kroman N. (2014). Prognosis of Synchronous Bilateral Breast Cancer: A Review and Meta-Analysis of Observational Studies. *Breast Cancer Research and Treatment*.

[5] Goldhirsch A., Winer E. P., Coates A. S. (2013). Personalizing the Treatment of Women With Early Breast Cancer: Highlights of the St Gallen International Expert Consensus on the Primary Therapy of Early Breast Cancer 2013. *Annals of Oncology*.

[6] Coates A. S., Winer E. P., Goldhirsch A. (2015). Tailoring Therapies—Improving the Management of Early Breast Cancer: St Gallen International Expert Consensus on the Primary Therapy of Early Breast Cancer 2015. *Annals of Oncology*.

[7] Baretta Z., Olopade O. I., Huo D. (2015). Heterogeneity in Hormone-Receptor Status and Survival Outcomes Among Women With Synchronous and Metachronous Bilateral Breast Cancers. *The Breast*.

[8] Renz D. M., Böttcher J., Baltzer P. A. T. (2010). The Contralateral Synchronous Breast Carcinoma: A Comparison of Histology, Localization, and Magnetic Resonance Imaging Characteristics With the Primary Index Cancer. *Breast Cancer Research and Treatment*.

[9] Mruthyunjayappa S., Zhang K., Zhang L., Eltoum I.-E. A., Siegal G. P., Wei S. (2019). Synchronous and Metachronous Bilateral Breast Cancer: Clinicopathologic Characteristics and Prognostic Outcomes. *Human Pathology*.

[10] Li X., Wang Y., Pan B. (2021). Clinical Characteristics and Clinicopathological Correlations of Bilateral Breast Cancer in China: A Multicenter Study From Chinese Society of Breast Surgery (CSBrS-006). *Chinese Journal of Cancer Research*.

[11] Sighoko D., Liu J., Hou N., Gustafson P., Huo D. (2014). Discordance in Hormone Receptor Status Among Primary, Metastatic, and Second Primary Breast Cancers: Biological Difference or Misclassification?. *The Oncologist*.

[12] National Comprehensive Cancer Network (2013). Breast Cancer, Version 3.2013: Featured Updates to the NCCN Guidelines [NCCN Guidelines]. https://www.nccn.org.

[13] Huang L., Liu Q., Lang G. T., Cao A. Y., Shao Z. M. (2020). Concordance of Hormone Receptor Status and BRCA1/2 Mutation Among Women With Synchronous Bilateral Breast Cancer. *Frontiers in Oncology*.

[14] Mejdahl M. K., Wohlfahrt J., Holm M. (2019). Breast Cancer Mortality in Synchronous Bilateral Breast Cancer Patients. *British Journal of Cancer*.

[15] Esclovon J. W., Ponder M., Aydin N., Misra S. (2016). Challenges of Treating Incidental Synchronous Bilateral Breast Cancer With Differing Tumour Biology. *Case Reports*.

[16] Aranda-Gutierrez A., Gomez-Picos A., Ferrigno A. S., Moncada-Madrazo M., Diaz-Perez H. (2020). Molecular Subtype Discordance in a Young Woman With Synchronous Bilateral Breast Cancer: A Case Report. *Cureus*.

[17] Arafah M., Kfoury H. K., Zaidi S. N. (2010). HER2/Neu Immunostaining in Invasive Breast Cancer: Analysis of False Positive Factors. *Oman Medical Journal*.

[18] Garrison Jr L. P., Babigumira J. B., Masaquel A., Wang B. C. M., Lalla D., Brammer M. (2015). The Lifetime Economic Burden of Inaccurate HER2 Testing: Estimating the Costs of False-Positive and False-Negative HER2 Test Results in US Patients With Early-Stage Breast Cancer. *Value in Health*.

[19] Dhadlie S., Whitfield J., Hendahewa R. (2018). Synchronous Bilateral Breast Cancer: A Case Report of Heterogeneous Estrogen Receptor Status. *International Journal of Surgery Case Reports*.

[20] Lower E. E., Glass E. L., Bradley D. A., Blau R., Heffelfinger S. (2005). Impact of Metastatic Estrogen Receptor and Progesterone Receptor Status on Survival. *Breast Cancer Research and Treatment*.

[21] Jiang Z., Wang H., Wang S. (2021). Chinese Expert Consensus Statement on the Clinical Diagnosis and Treatment of Breast Cancer Bone Metastasis and Bone Related Disease. *Translational Breast Cancer Research*.

[22] Eisen A., Somerfield M. R., Accordino M. K. (2022). Use of Adjuvant Bisphosphonates and Other Bone-Modifying Agents in Breast Cancer: ASCO-OH (CCO) Guideline Update. *Journal of Clinical Oncology*.

[23] Wang X., Xue Y. (2023). Clinicopathological Characteristics and Prognostic Analysis of Breast Cancer With a Hormone Receptor Status of ER(-)/PR(+). *Frontiers in Endocrinology*.

[24] Johnston S. R. D., Toi M., O'Shaughnessy J. (2023). Abemaciclib Plus Endocrine Therapy for Hormone Receptor-Positive, HER2-Negative, Node-Positive, High-Risk Early Breast Cancer (monarchE): Results From a Preplanned Interim Analysis of a Randomised, Open-Label, Phase 3 Trial. *Lancet Oncology*.

[25] Mallio C. A., Greco F., Gaudino F., Beomonte Zobel B., Quattrocchi C. C. (2023). Computed Tomography Density Changes of Bone Metastases After Concomitant Denosumab. *Skeletal Radiology*.

[26] Fountzilas E., Kotoula V., Zagouri F. (2016). Disease Evolution and Heterogeneity in Bilateral Breast Cancer. *American Journal of Cancer Research*.

[27] Prabhu J. S., Wahi K., Korlimarla A. (2012). The Epigenetic Silencing of the Estrogen Receptor (ER) by Hypermethylation of the ESR1 Promoter Is Seen Predominantly in Triple-Negative Breast Cancers in Indian Women. *Tumor Biology*.

[28] Copur M. S., Ramaekers R., Gauchan D., Crockett D., Clark D. (2017). Synchronous Bilateral Breast Cancer With Discordant Histology. *Oncology (Williston Park, NY)*.

[29] Hayashi M., Yamamoto Y., Takata N., Iwase H. (2013). A Case of Synchronous Bilateral Breast Cancer With Different Pathological Responses to Neoadjuvant Chemotherapy With Different Biological Character. *Springerplus*.

[30] Karsten M., Stempel M., Radosa J., Patil S., King T. A. (2016). Oncotype DX in Bilateral Synchronous Primary Invasive Breast Cancer. *Annals of Surgical Oncology*.

[31] Aranda-Gutierrez A., Ferrigno A. S., Vaca-Cartagena B. F. (2021). 179P Discordance Rates of Clinicopathological Features in Bilateral Breast Cancer. *Annals of Oncology*.

